# Risk prediction of healthcare-associated neonatal sepsis in Amhara, Ethiopia: A prospective cohort study

**DOI:** 10.1016/j.gpeds.2025.100268

**Published:** 2025-09

**Authors:** Nadine Najjar, Abebe Gebremariam Gobezayehu, Joseph Hopkins, Heran Biza, Habib Yakubu, Lindsay Denny, Mulusew Lijalem, Lamesgin Alamineh Endalamaw, Yakob S. Ahmed, Taye Zeru Tadege, Mekuanint Wasihun Endalew, Yichalal Endayehu Tafere, Gurmesa Tura Debelew, Erin Stone, Christine L. Moe, John N. Cranmer

**Affiliations:** a Department of Pediatrics, Emory University, Atlanta, Georgia, United States; b Emory Ethiopia, Amhara Regional Office, Bahir Dar, Ethiopia; c Independent Consultant, Atlanta, Georgia, United States; d Center for Global Safe Water, Sanitation, and Hygiene at Emory University, Atlanta, Georgia, United States; e UNICEF, New York City, New York, United States; f ReachAnother Foundation Ethiopia; g Amhara Public Health Institute, Bahir Dar, Ethiopia; h Debre Tabor General Hospital, Bahir Dar, Ethiopia; i Felege Hiwot Referral Hospital, Bahir Dar, Ethiopia; j Emory University Rollins School of Public Health, Atlanta, Georgia, United States; k Emory University Nell Hodgson Woodruff School of Nursing, Atlanta, Georgia, United States

## Abstract

**Background:**

Neonatal sepsis is a major contributor to neonatal mortality in low- and middle-income countries. Globally, rapid diagnosis and treatment are often a challenge, and standard antibiotic therapy is threatened by antimicrobial resistance (AMR). This study quantifies the predictors of healthcare-associated neonatal sepsis in Amhara, Ethiopia.

**Methods:**

This prospective cohort study recruited normal and low birth weight (LBW) neonates from two hospitals. Neonates with suspected healthcare-associated sepsis had blood cultures drawn to identify the organisms and AMR patterns. We used univariable and multivariable logistic regression to determine risk factors for culture-confirmed sepsis. Next, we performed marginal effects analysis to create a clinical tool for prospectively measuring newborn sepsis risk.

**Results:**

Twenty percent of 605 neonates developed healthcare-associated, culture-confirmed sepsis. 44.9% were resistant to first-line empiric therapy, and only 4% of those who failed first-line drugs had sepsis organisms susceptible to second-line drugs. Multivariable logistic regression identified five primary predictors: LBW (aOR 3.4), twin birth (aOR 4.7), maternal history of preterm births (aOR 2.7) or history of LBW births (aOR 2.6), low family income (aOR 1.7), and birth at the general hospital (aOR 2.5). There were significant differences in sepsis and mortality by facility and birthweight.

**Conclusions:**

Neonatal sepsis and AMR pose significant risk to our study population. Due to the high proportion of AMR and likelihood of treatment failure, developing effective facility-based sepsis prevention strategies is an urgent priority for advancing newborn survival in Amhara, Ethiopia and in similar global contexts.

## Introduction

Despite significant strides in reducing under-five mortality globally, neonatal mortality has not declined as quickly. Neonatal mortality contributed to 47% of all deaths among children under five years of age between 1990 and 2019.^1^^,^^2^ Further, reductions in the neonatal mortality ratio in sub-Saharan Africa have stalled compared to other global contexts^2^^,^^3^ and neonatal infections are a primary driver of neonatal mortality. Sepsis, meningitis, and pneumonia are implicated in nearly one third of all global newborn deaths^1^^,^^2^, and three million newborns globally experience sepsis each year.^4^ In low- and middle-income countries (LMICs), the sepsis incidence is 166 infections per 1000 live births^5^. Many LMICs, including Ethiopia, have substantially reduced overall under-five mortality in the Sustainable Development Goal (SDG) era^1^. Yet, Ethiopia’s neonatal mortality ratio has only declined by 15% since 2005^6^. Ethiopia’s national ratio of 33 deaths per 1000 live births remains largely driven by infections and co-occurring low birth weight (LBW).^4^^,^^5^

Neonatal sepsis is defined as systemic infection from bacteria, viruses or fungi in newborns up to 28 days of life^7^. Early-onset sepsis (EOS) is generally attributed to infections acquired through vertical transmission from the mother during birth and presents within the first three days of life. Late-onset sepsis (LOS) occurs after 3 days of life (or 7 days by some definitions) and is generally attributed to infections from the surrounding environment (such as the healthcare environment).^7^ LBW and premature infants are particularly susceptible to both early- and late-onset sepsis^7^. Traditionally, healthcare-associated infections are defined as infections presenting more than 48 hours after hospitalization. In the context of LMICs, some scholars assert that any infection acquired in a hospital-born baby should be define as healthcare-associated.^8^

Neonatal sepsis is most often diagnosed through a combination of clinical signs and symptoms of infection or inflammation and/or isolation of a causal organism from sterile body fluids such as blood or cerebrospinal fluid.^9^ The organisms that cause sepsis differ across context (home versus hospital) and geographic settings. Globally, early onset sepsis (EOS) is most often caused by *Escherichia coli* and *Streptococcus agalactiae* (or Group B streptococci)^7^. However, neonatal sepsis etiologies in LMICs include a wider variety of gram negative (e.g., *Klebsiella pneumoniae, Escherichia coli* (*E. coli), Pseudomonas* species, *Acinetobacter* species) and gram positive bacteria (e.g., *Staphylococcus aureus (S. aureus)* and Group B Streptococci*)*^8^^,^^10^. In Sub-Saharan Africa, gram-negative bacteria, such as *E. coli* and *Klebsiella* species, and gram-positive bacteria, such as *S. aureus*, are more common^10^^,^^11^. In this context, gram negative organisms are the dominant drivers of EOS, while gram-positive species more commonly drive infections in later in infancy.^10^

In many LMIC healthcare facilities, neonatal sepsis treatment is empiric since many facilities don not have the laboratory capacity to run blood cultures and sensitivity analyses. Currently, the World Health Organization (WHO) recommends empiric treatment with gentamicin plus ampicillin (or penicillin) as first line drugs in LMIC facilities when culture and sensitivity assays are not available.^12^ However, antimicrobial resistance (AMR) is now a leading global threat to health. AMR may be driven by multiple factors including environmental cleanliness, hygiene conditions (e.g, clean water access), practice (e.g., hand washing) and antibiotic misuse.^13^ While the WHO provides guidelines for treatment of neonatal sepsis, recent studies show these recommendations may be inadequate our outdated based on the emerging research on the growing burden of AMR.^14^^,^^15^ A recent modeling study suggests the globe is already facing a high AMR burden. Further, 5.0 million global deaths were associated with AMR and 1.3 million were directly attributable to AMR.^16^ In neonates specifically, data on resistance from multiple global contexts indicate up to 70% of healthcare-associated sepsis in LMICs may not be susceptible to WHO’s suggested first-line regimen (ampicillin and gentamicin).^8^ In a systematic review of community-acquired infant sepsis, only 43% of bacterial isolates were not susceptible to WHO first-line treatment. Although some global facilities have access to third generation cephalosporin (e.g., ceftazidime) for second-line therapy, adding ceftazidime did not provide treatment benefit in global studies.^17^

Consequently, the primary objectives of this study are to: 1) measure the burden of healthcare-associated neonatal sepsis at two hospitals in the Amhara Region of Ethiopia; 2) identify clinical, maternal and sociocultural risk factors predictive of neonatal sepsis and 3) measure the differential burden of sepsis and mortality by health facility and birth weight.

## Materials and methods

### Study design, facilities, and participant selection

This prospective cohort study was conducted at two public healthcare facilities in the Amhara region of Ethiopia as part of a WHO multi-site implementation trial to design and test strategies that increase Kangaroo Mother Care (KMC) uptake, efficacy, and impact.^18^, ^19^, ^20^ One was a large, urban referral hospital with 450 monthly deliveries. The second was rural and had 260 births per month. The study in this manuscript recruited infants born at either hospital who weighed <2000 grams at birth (defined as Low birth weight, LBW) and ≥2000 grams at birth (defined as Normal Birth Weight, NBW). Every <2000-gram infant and every fourth infant ≥2000 grams were recruited. After newborns birthed in the facility were weighed, they were recruited shortly after birth by study staff at each hospital. All newborns received care in either the post-natal ward, Kangaroo Mother Care ward (KMC) or neonatal intensive care unit (NICU). Participants were recruited between 22/10/2018 through 27/06/2019. Infants who were not born at one of the study hospitals, or whose parents did not provide consent, were excluded from the study.

### Data collection

After obtaining study consent, trained data collectors obtained familial, socio-demographic, maternal, and clinical data using standardized questionnaires. The data collectors captured baseline and clinical information using REDCap on mobile tablets.^21^^,^^22^ Data were then exported as .CSV files and converted to .DTA files for analysis in STATA-MP (version 17 for Mac, College Station, Tx, USA). Clinical blood specimens and baseline clinical data were merged in open-source *pandas* Python Data Analysis library. Multiple databases (baseline, lab sample, and follow up) were cleaned, reconciled, and linked based on participant identification number into a single database for analysis.

Infants were followed during their hospital stay. Neonates received standard sepsis care at both facilities overseen by the hospital clinicians. However, any infant with signs or symptoms of clinical suspected sepsis had blood samples drawn for culture and sensitivity. The samples were drawn at the respective facility and tested at the Amhara Public Health Institute (APHI) internationally certified laboratory. Symptom, health outcome, and household data were collected by in-person or telephone interview at 7 and 14 days post-discharge and Day 28 of life. This manuscript reports the incidence and covariate predictors of healthcare-associated sepsis during admission. Healthcare-associated neonatal sepsis was defined as bloodstream infection diagnosed following birth and inpatient stay at a study hospital. First line empiric therapy is defined as either ampicillin or penicillin plus gentamicin. Second line therapy is defined as ampicillin and ceftazidime, based on the drugs available in the Ethiopian government system and Ministry of Health guidelines for neonatal sepsis.

### Variables

The primary outcome was the incidence of any neonatal sepsis confirmed by laboratory culture. Secondary outcomes include antimicrobial resistance (AMR) and mortality. Microbiological testing of blood specimens from neonates with signs and symptoms of sepsis is the intended standard of care for public hospitals when lab supplies are available. Blood samples were taken by doctors or nurses from any newborns in NICU or KMC units who were suspected to have sepsis within the first 28 days of life. Blood culture supplies were provided for all suspected cases of sepsis by this study.

### Laboratory methods

All samples were analyzed at the Amhara Public Health Institute (APHI), an ISO certified laboratory (Standard 15189), to determine the causal organism(s), and antimicrobial susceptibility. The blood samples were dispensed into broth media containing 0.025% SPS. Blood culture vials were incubated at 35-37°C for 7 days and are examined daily for the presence of growth. Vials were sub-cultured onto MacConkey and Blood agar plates after 24 hours incubation and/or when growth was apparent, and at the end of the 7-day incubation period. Sub-culture plates were incubated at 35 –37°C in 5% CO_2_ and examined for growth after 24 hours of incubation. Bacterial isolates were identified and tested for antimicrobial susceptibility using VITEK 2-Microbial ID—Susceptibility testing system (Biomerieux, Marcy-l'Étoile, France). Results from the microbiological testing were input into the laboratory’s database as well as shared with the study team and hospital clinicians.

### Analytic methods

We predicted culture-confirmed antimicrobial resistance (AMR) using the blood samples of newborns with culture-confirmed sepsis with multivariable logistic regression. Our secondary outcomes include antimicrobial resistance incidence and mortality. To predict AMR, we used socio-demographic, clinical, obstetric, and maternal-family variables. We used a six-part method to generate and test the final MLR and produce a sepsis risk algorithm for clinical practice using marginal effects.

### Patient characteristics and clinical outcomes

First, we described sample characteristics by hospital (general vs. referral) and birthweight category (low birth weight, <2000 g (LBW) versus normal birth weight >/=2000 grams). We examined four categories of sample characteristics at baseline—a clinical, social, familial, and space-time factors. Continuous variables were described using medians and interquartile ranges (IQR), and differences by birthweight and healthcare facility were compared using the Wilcoxon Rank Sum test. Categorical variables were described using percentages, and differences by facility and birthweight were compared using the Chi-squared test of independence (or Fisher’s Exact test for cell counts <6). Key clinical outcomes, such as sepsis, AMR, and mortality, were described using rates or proportions.

Second, based on theoretical factors and/or empiric distributions and frequencies for each predictor, we recategorized select predictors to optimally fit MLR models. For example, we modelled birthweight in several categories and used the cut-off of <2000 grams versus ≥2000 grams for the final multivariable model based on empiric distributions/frequencies and theoretical rational.

### Sepsis prediction: logistic regression

Third, we tested each of the variable’s ability to predict AMR using univariate logistic regression (ULR). For predicting sepsis, only the first culture-positive sample was included if patients had more than one positive blood culture over time. Fourth, we created a multivariable logistic regression (MLR) model with the highest overall area under the curve (AUC), where all predictors had a high effect size (high or low adjusted odds ratio, aOR) and were statistically significant at p</=0.05. We used a variable-by-variable reduction method to create the MLR where predictors with the highest p-value and/or lowest effect size (low or high odds ratio) were removed one-by-one from the initial MLR with all predictors. Further, we preemptively removed variables that were empirically or theoretically co-linear. For example, zone, region, and hospital were closely related based on a geographic health system, so we only tested hospital. Also, we created certain interaction terms where variables were both clinically relevant but highly related (e.g., birthweight and multiple births).

In addition, we tested the impact of removing the variable on the AUC for the MLR model to ensure that predictive variables were not inadvertently removed using the significance and effect size criterion.

### Model performance

Fifth, after producing the final MLR, we performed post-model checks. The overall performance was measured using the area under the receiver operating characteristics curve (AUC). We also confirmed the overall MLR and individual variables did not have a variance inflation factor (VIF) >/= 10 to ensure no colinear variables were present in the model. This resulted in a MLR model which retained predictors that had a high effect size and statical significance while preserving the highest possible AUC. The resulting MLR was deemed the “gold model” for sepsis prediction.

### Clinical algorithm: marginal effects

The final “golden” MLR was used to generate a real-world algorithm for prospectively predicting individual-level sepsis risk for future patients using as-observed marginal effects. Individual risk scores for each combination of patient variables from the final MLR model are reported as the percent risk of for developing hospital acquired, sepsis-confirmed newborn sepsis.

### Ethics

Ethical approval was obtained from the Amhara Public Health Institute Ethics Review Committee (#APH-IHR-TD-2010108-GC on June 1, 2016) and the Emory University Institutional Review Board (AM2_IRB00089275 on April 27, 2018). We obtained informed, written consent from one of the parents of each newborn participant. For parents who did not read and write, they provided a thumb print which was attested by a signed witness.

## Results

### Patient characteristics

Clinically, there were some socioeconomic, clinical and differences between the two hospitals. However, at both facilities, the median maternal age of 27 was similar (IQR=24-30; p=0.229), and median age gap between parents was 5 years (IQR=3-7, p=0.071). Clinically, the referral hospital had a higher proportion of LBW newborns (25.8% versus 13.4%, p=0.002, supplementary Table 1), higher proportion of completed ANC visits at 77.7% (versus 53.9%, p <0.001), and twice as many previous LBW newborns (20.6% versus 10.9%, p=0.005). From a socioeconomic perspective, families at the referral hospital had double the annual household income (40,000 versus 22,000 birr, p=0.001), double income per household member (14,000 versus 7,500 birr, p <0.001), and were twice as likely to own homes (91.2% versus 59.5%, p <0.001) and cell phones (96.5% versus 53.4%, p <0.001). Mothers at the referral hospital were twice as likely to complete secondary school (10.6% versus 4.1%, p <.0.001) and report reading/writing skills (80.0% versus 40.6%, p <0.001). Furthermore, double the 20.2% families at the general hospital did not have any parent who was literate compared to 12.0% at the referral hospital (p=0.001).

### Clinical outcomes and resistance patterns

The overall incidence of hospital-acquired, culture-confirmed neonatal sepsis was 20% in the entire population (Table 1). This risk was 2.5 times higher in LBW newborns compared to NBW infants (LBW=38.1%, NBW=15.4%, p=0.007). Further, 25.1% of newborns at the general hospital developed sepsis compared to 15.6% at the referral hospital.

**Table 1:** Health outcomes by birthweight and facility at 7 days post-discharge.

**Outcomes**		**Birthweight** (LBW <2000 g)		**Facility**	
	**Total (n=593)**	**Normal (n=475)**	**Low** **(n=118)**	**Referral (n=327)**	**General (n=266)**
% Culture Positivity (Among all newborns, percentage)	19.9	15.4	38.1	15.6	25.1
Sepsis-associated mortality (Among all septic newborns, percentage)	6.7	0	18.4	15.6	0
**Antimicrobial Resistance**					
% Antimicrobial resistance (AMR, Among all septic infants)	86.4	84.9	88.9	94.1	80.6
AMR, 1^st^ line empiric therapy, Any drug	44.9	39.7	53.3	56.8	35.8
AMR, 1^st^ line empiric therapy, Both drugs	25.4	27.4	22.2	37.3	16.4
AMR, 2^nd^ Line empiric therapy, Any drug	43.2	38.4	51.1	52.9	35.8
AMR, 2^nd^ line empiric therapy, Among resistant to *any* 1^st^ line drug	96.2	96.6	95.8	93.1	100.0

Healthcare-associated neonatal sepsis was associated with mortality. There was a 6.7% overall sepsis-associated mortality (Table 1). However, neonates only died at the referral hospital (15.6%). Moreover, this differed dramatically by birthweight as well, with LBW neonates bearing 18.4% sepsis-associated mortality burden compared to no risk in the NBWs.

Overall, over 80% of clinical isolates from sepsis cases exhibited resistance to one or more antibiotics. AMR was higher at the referral hospital and impacted 94% of septic newborns (Table 1). Dramatically, nearly half had resistance to standard first line therapy available at the facilities (45%). Of the resistant isolates, only 4% would be susceptibility to second line therapy available at the facilities. This AMR resistance to first line therapy differed moderately by birthweight (53.3% for LBW versus 39.7%) and facility (56.8% at referral hospital versus 35.8%). The sample contrast between facilities was greatest when comparing resistance to both first-line drugs, with 37.3% of isolates resistant to both drugs at the general hospital versus 16.4% at the referral hospital.

Table 2 stratifies sepsis incidence by by birth weight and facility. Overall, 50.0% of sepsis cases were associated with gram negative organisms. *Klebsiella* species were the most commonly identified organisms (25.4%) and were more common at the referral hospital (35.3% versus 17.9%, p=0.032). *E. coli* and all *Serratia* species were the second most common organism at 5.1% each. There were no significant differences by birthweight or facility. Gram positive organisms were isolated from 48.3% of sepsis samples; this did not differ by birthweight or facility. The primary gram positive organisms were *Staphylococcus aureus* (29.7%) and CoNS (20.3%). *Staphylococcus aureus* was marginally more common at the general hospital (35.8% versus 21.6%, p=0.093) while CoNS was nearly three times as likely at the referral hospital (31.4% versus 11.9%, p=0.009).

**Table 2:** Bacterial isolates from neonatal sepsis cases by facility and birthweight.

		**Birthweight** (LBW <2000 g)				**Facility**		
	*Total (%)* *(n=593)*	*Normal (%)* *(n=475)*	*Low (%)* *(n=118)*	*p-*1	*Referral (%)* *(n=327)*		*General (%)* *(n=266)*	*p-*1
**Sepsis Indicators**								
Culture Confirmed Sepsis	19.9	15.4	38.1	0.000^2^		15.6	25.1	0.005^2^
Polymicrobial Sepsis	9.3	8.2	11.1	0.746		15.7	4.5	0.054
**Gram Negative**								
All Gram (-) Organisms	50.0	43.8	60.0	0.088^2^		52.9	47.8	0.577^2^
*Klebsiella* species *(including K. pneumoniae)*	25.4	24.7	26.7	0.808^2^		35.3	17.9	0.032^2^
*Escherichia coli*	5.1	2.7	8.9	0.140		5.9	4.5	1.000
*Serratia* species *(with Serratia proteamaculans)*	5.1	5.5	4.4	1.000		2.0	7.5	0.233
Other gram negatives^3^	15.3	11.0	22.2	0.098^2^		9.8	19.4	0.151
**Gram Positive**								
All Gram (+) Organisms	48.3	53.4	40.0	0.156^2^		43.2	53.7	0.176^2^
*Staphylococcus aureus*	29.7	32.9	24.4	0.330^2^		21.6	35.8	0.093^2^
Coagulase negative *Staphylococcus* species	20.3	23.3	15.6	0.311^2^		31.4	11.9	0.009^2^
All other gram positives 4	6.8	5.5	8.9	0.478		1.2	10.5	0.135

1 Differences between groups were compared with the Fischer’s exact test for groups with cell counts ≤6

2 For larger groups, we compared differences with the Chi-square test of impedance

3 All gram negatives except Serratia species, klebsiella and E. coli

4 Excluding S. aureus and CoNS

### Sepsis prediction

Next, we used univariable and multivariable logistic regression to identify key factors that predicted sepsis. These factors were examined in five categories—clinical, obstetric, social, parental, and place-time (e.g., facility). Among the four clinical factors, birthweight <2000 grams was the greatest risk factor for sepsis with a 3.4-fold odds (p=0.000, Table 3). Lack of antenatal care carried 2.8-fold odds of sepsis (p=0.000), followed by multiple births for the current pregnancy (odds ratio (OR)=2.7, p=0.010). There was a strong dose-response relationship when testing the interaction of birthweight and multiple births with sepsis risk with a high Area Under the Curve (AUC) of 61.5% for this single variable. Among normal birthweight (NBW) multiple births, the odds of sepsis were 1.1 and not significant (p=0.938). Sepsis odds increased to 3.1 for LBW singletons (p=0.000) and was higher at 4.1 for LBW multiples (p=0.001). Among six obstetric history variables, there was increasing odds of the current neonate having sepsis as the mother’s number of previous preterm births and LBW births increased. Mothers who had previously delivered two or more preterm infants had 5.4-fold odds of current newborn sepsis compared to those with no previous pre-terms (p <0.001). The magnitude of risk was similar among mothers with ≥2 previous LBW infants (OR=4.8, p=0.001). Among five social factors, the greatest risk of sepsis was among infants born in South Gondar (OR=2.2, p=0.001)—the location of the general hospital (Table 1). Next, as family income in quartiles decreased to quartiles 1-2, sepsis risk increased (OR=1.7, p=0.016, compared to quartiles 3-4). Among the parent factors, the greatest sepsis risk was among neonates with two illiterate parents (OR=2.2, p=0.001) compared to neonates with 1 or 2 literate parents. Maternal and paternal illiteracy were both strong independent predictors (maternal illiteracy=1.8, p=0.019; paternal=1.9, p=0.005). Finally, in the place-time category, we modelled the impact of hospital facility on sepsis risk. Neonates at the general hospital had a 2.5-fold risk compared to the referral hospital (p=0.007).

**Table 3:** Sepsis prediction with univariate logistic regression.

**Predicting Sepsis with Univariate Logistic Regression**			
**Sepsis Predictors by Category**			
**Place-Time Factors**	**OR**^1^	**p-value**	**AUC**^2^
Primary Hospital (Ref: Referral hospital)	2.5	0.007	60.9
**Clinical Factors**			
Birthweight & Facility interaction (Ref: NBW & Referral Hospital)			
Low birth weight (LBW <2000 g), Referral	1.5	0.385	65.6
NBW & General Hospital	2.1	0.061	
LBW & General Hospital	19.9	0.005	
Birthweight and Multiple births interaction			
Normal birthweight, multiples	1.1	0.938	61.5
Low birth weight (LBW <2000 g), singleton	3.1	0.000	
LBW, multiples	4.3	0.001	
Birthweight			
Low Birth Weight (Ref: ≥ 2000 g)	3.4	0.000	61.4
Multiple births ≥1 (Ref: Singleton)	2.7	0.010	53.1
No Antenatal care (Ref: ≥1)	2.8	0.028	52.1
Assisted Delivery, Forceps or Caesarean Section (Ref: normal vaginal)	0.4	.237	51.2
**Obstetric History Factors**			
Total pregnancies (Gravida)	0.9	0.131	54.0
Previous preterm births (Ref: 0) 1	2.7	0.000	60.1
≥2	5.4	0.000	
Previous number of LBW in categories (Ref: 0) 1	2.6	0.000	60.4
≥2	4.8	0.001	
Total living children	0.9	0.406	52.3
Total previous births	0.9	0.256	52.7
History of any deceased newborns (Ref: None)	1.4	0.424	50.1
**Social Factors**			
Geography-Zone^4^ (Ref: Bahir Dar City)			
South Gondar	2.2	0.001	59.1
West Gojam	1.4	0.271	
Household size ≥3 people (Ref: 1-2)	1.4	0.108	54.0
No Home ownership (Ref: Yes)	1.5	0.074	53.9
Cell Phone Ownership (Ref: Not owned)	1.3	0.279	52.4
Family income			
Quartiles 1-2 (Ref: Quartiles 3-4)	1.7	0.016	56.5
**Parent Factors**			
Maternal age category (Ref: 21-34 years)			
<20	1.1	0.7689	52.1
≥35	0.7	0.278	
Maternal education, Secondary or higher (Ref: Primary or less)	1.7	0.038	56.6
Mother not literate (Ref: literate)	1.8	0.019	55.9
Father not literate (Ref: literate)	1.9	0.005	55.9
No parents literate (Ref: 1 or 2 parents literate)	2.2	0.001	56.1
Parent age gap across 4 categories^3^	1.5	0.006	59.1

1 Odds Ratio

2 Area Under the Curve

3 Parent age gap (Father (-) Mother age in years): ≤ (-)1 - 2, 3-5, 6-10, ≥11 (consider ≤5 & >6)

4 A zone is the second highest level of administrative structure after a region. The average population of a zone in Amhara region is about 1.9 million. Each administrative zone on average has about 12 districts/woredas under it. Each woreda/district has an estimated population of 160,000.

Based on these univariable predictors, we created a multivariable logistic regression (MLR) model to predict healthcare-associated neonatal sepsis. In addition, we compared the final MLR predicting sepsis to MLR models for each of the five categories of predictors (clinical, obstetric history, social, parent, place-time) and to a comprehensive MLR model with all variables (Tables 4.1 and 4.2). The final model had one place-time, one clinical, and two social variables. To further delineate the relationship between birthweight and place-time exposure, both variables were combined to compare LBW and NBW newborns born at either facility. In the adjusted model, the birthweight and the birth facility conveyed the highest sepsis risk with a 24.0-fold increased risk at the general hospital for LBW infants (p=0.003), compared to a 2.0-fold increased risk at the referral hospital for LBW infants (p=0.179). Next, owning a cell phone was associated with an increased sepsis risk of 2.6 (p=0.049) and low household income in quartiles 1-2 also conveyed a 2.5-fold increased risk (p=0.023) compared to quartiles 3-4. Although the interaction of birthweight with multiple births was a powerful predictor in the univariable model, it was not statistically significant in all categories in the progressively built multivariable model or when replacing categorical LBW ((p=0.537, 0.115, 0.044). However, the AUC were comparable between the final model and this MLR for the birthweight-multiples interaction (73.8%).

**Table 4.1:** Multivariate logistic regression model predicting newborn sepsis.

	**Variable Performance**			**Model Performance**		
**Predictors**	**aOR**^3^	**p-value**	**VIF**^4^	**AUC**^5^	**AIC**^6^	**HL GOF**^7^
Hospital x Birthweight^1^ (Ref: Referral & Normal Birthweight)				72.9	178.2	0.2287
Referral & LBW	2.0	0.179	1.7			
General & NBW	3.0	0.020	2.6			
General & LBW	24.0	0.003	1.6			
Owns cell phone (Ref: Does not own cell phone)	2.6	0.049	5.2			
Low Household Income, Quartiles^2^ 1-2 (Ref: Quartiles 3-4)	2.5	0.023	2.7			

1 Normal Birthweight (NBW, ≥2000 grams); Low birthweight (LBW, <2000 grams)

2 Household income quartiles: Q1: 0-19,999 birr, Q2: 20,000-29,999, Q3: 30,000-45,999, Q4: 46,000-150,000

3 Adjusted Odds Ratio

4 Variance inflation factor (VIF, uncentered); mean VIF=4.2

5 Area Under the Curve

6 Akaike Information Criterion

7 Homer Lemeshow goodness-of-fit

**Table 4.2:** Sepsis model performance for 5 categories of variables, a comprehensive model and final model with all significant predictors.

**Models**	**Place-Time**	**Social**	**Parent**	**Clinical**	**Obstetric**	**Comprehensive^4^**	**Final**^5^
AUC^1^	60.9%	65.4%	64.1%	65.3	64.3%	84.1%	72.9%
AIC^2^	223.5	491.9	349.5	553.2	564.2	137	178.2
HL GOF^3^	–	0.2584	0.1686	0.4507	0.9665	0.2541	0.7376

^1^Area Under the Curve

^2^Akaike Information Criterion

^3^Homer Lemeshow goodness-of-fit

^4^All variables from all five categories of predictors tested together regardless of statistical significance; includes all variables from Place-Time, Social, Parent, Clinical & Obstetric categories

^5^Final MLR model where all predictors were statistically significant at p<0.05

The final MLR predicting sepsis had a high overall predictive performance with an AUC of 72.9%, and this was higher than the AUC for the four other MLR by factor (place-time=60.9%, social=64.5%, parent=64.1%, clinical 65.3%, obstetric=64.3%). The Hosmer-Lemeshow goodness-of-fit test of p=0.229 met the criterion for using this model. This model’s Akaike information criterion (AIC) was 178.2 and was lower than all other MLR models for the four factors (range=223-564). In addition, there was no indication of co-linear predictors with variable inflation factors (VIFs) with a mean VIF of 4.2.

### Algorithm for sepsis risk stratification

The clinical prediction tool was developed to prospectively estimate sepsis risk for future neonates in Amhara to strategically target care. The final multivariable model included facility, birthweight, cell phone ownership, and household income in quartiles. Based on marginal effects analysis, the risk of sepsis acquisition varied greatly from 20.0% to 97.5% using these four characteristics (Table 5). Infants born at the referral hospital who were NBW (>2000 g) to families who owned a cell phone and had a higher income (quartile 3-4, ≥30,000 birr / month) had the lowest sepsis risk at 20.0%. In contrast, 97.5% of all newborns who contracted healthcare-associated sepsis were born with LBW at the general hospital to a lower income family (quartile 1-2, 0-29,999 birr/month) that did not own a cell phone. Additionally, the risk difference between newborns born at the general and referral hospital varied based on the presence or absence of the included risk factors. For example, for newborns born to families with higher income and cell phone ownership, the risk difference between NBW and LBW newborns varied significantly by facility. However, when comparing newborns from lower income background and no cell phone ownership, the risk difference was unchanged for NBW and LBW infants (Table 5). Although overall sepsis risk was 20.0% among all birthweights at all facilities, there was substantial variability by birthweight and facility.

**Table 5:** Clinical prediction of sepsis by facility and risk factor: marginal effects analysis.

**Risk Factors Present**			**% Sepsis Risk by Hospital** (p-value)		
**Birthweight**	**Own Cell Phone**	**Income**	**Referral**	**General**	**Risk Difference** (General -Referral)
**Normal**	No	Higher	**20.0** (0.042)	**43.1** (0.001)	23.1
**Low**			**32.9** (0.012)	**85.7** (0.000)	52.8
**Normal**	No	Lower	**38.6** (0.012)	**65.5** (0.000)	26.9
**Low**			**55.2** (0.000)	**93.8** (0.000)	38.5
**Normal**	Yes	Higher	**39.6** (0.000)	**66.5** (0.000)	26.9
**Low**			**56.3** (0.000)	**94.0** (0.000)	37.7
**Normal**	Yes	Lower	**62.2** (0.000)	**83.3** (0.000)	21.0
**Low**			**76.4** (0.000)	**97.5** (0.000)	21.0

NBW: Normal Birthweight, ≥2000 grams; LBW: Low Birthweight <2000 grams

Higher income: quartiles 3-4 (≥30,000 birr/month)

Lower income: quartiles 1-2 (0-29,999 birr/month)

## Discussion

This study highlights the threat of healthcare-associated neonatal sepsis and AMR to newborn and under-five survival in contexts such as Amhara, Ethiopia. Most notably, the sepsis incidence was alarmingly high at 199 per 1000 births; the greatest risk was among LBWs (381 per 1000 births compared to 154 per 1000 for NBW births). These findings exceed previously reported average incidence of 166 per 1000 births LMICs by 130%.^5^ The prevalence of AMR sepsis was profoundly high with 86% of culture isolated organisms resistant to at least one antibiotic. Perhaps most concerning is that only 4% of infants with organisms resistant to first-line treatment could be treated by second-line drugs. Consequently, among healthcare-associated neonatal sepsis cases that fail first-line empiric therapy, there are essentially no effective therapeutic options at government hospitals (except at the few facilities nationally with 3^rd^ line vancomycin).

Our results present novel information on healthcare-associated neonatal sepsis in Ethiopia and corroborate previously cited sepsis incidence in sub-Saharan Africa. A systematic review of neonatal sepsis prevalence in Ethiopia identified a national prevalence of 45.0%. This study included both healthcare- and community-associated sepsis. Community- and Facility-sepsis prevalence varied by region, and was highest in Amhara at 64.0% and lowest in Southern Nations, Nationality, and People region at 28.0%.^23^ While sepsis prevalence in this population is comparable to previously cited numbers,^23^ prevalence of AMR-associated sepsis exceeds estimates from prior studies. For example, Downie et al previously showed susceptibility to first-line antimicrobials reached 57% of all neonates with bacteremia.^17^ Further, LBW and preterm infants were at higher risk of sepsis compared to normal birth weight newborns (OR 1.42) (95% CI, 1.07, 1.88).^24^ All-cause mortality among preterm infants is very high and ranges from 23-29% in Ethiopian NICUs,^25^ with mortality increasing as gestational age and birth weight decrease, consistent with the findings in our study.

The care environment may play a critical role in neonatal sepsis and mortality. A recent multi-country review focused specifically on healthcare-associated neonatal infections, showing three to twenty times higher incidence in LMICs compared to high-income countries (HICs).^8^ LMIC facilities can have limited sepsis surveillance data—particularly outside of large urban centers^26^. However, the overall prevalence of healthcare-associated infection among all age groups is estimated 15.5 per 100 patients in LMICs^27^. This is especially striking when compared to Europe, where estimated prevalence of healthcare-associated infection is three-fold lower at 4.5 per 100 patients.^28^ There are limited data on the quantitative relationships between specific hospital characteristics, incident neonatal sepsis and AMR in published literature. Consequently, this study’s insights on care environment add new insights to scholarship on AMR risk predictors globally.

A critical task in preventing healthcare-associated neonatal sepsis is determining at-risk populations. Using multivariable logistic regression, this study identified the risk factors most likely to contribute to a sepsis diagnosis. In this study, LBW infants experienced more than double the incidence of sepsis compared to NBW infants. Further, our findings that prior preterm births are a risk factor for neonatal sepsis, is consistent with prior literature^24^*.* However, our findings have identified other major concerns. For example, the multivariable analysis indicated that the birth facility played a major role in healthcare-associated sepsis risk, especially in the LBW population. There was a 12-fold increased sepsis risk in LBW newborns at the general hospital compared to the referral hospital. In context, however, this finding is nuanced because the proportion of AMR sepsis (94.1% vs. 80.6%) and sepsis-associated mortality (15.6% vs. 0%) were much higher at the referral hospital. These findings also indicate a dose-response interaction between LBW and multiple births for predicting sepsis risk in the univariable analysis. LBW and multiple births often co-occur. The multivariable model results suggest the joint interaction of LBW and multiple births may need to be considered as a powerful sepsis risk predictor in larger, prospective samples—particularly since this interaction had a profoundly high univariable AUC (61.5%)—the highest AUC among all single variables in univariable analysis (Table 3). Interestingly, cell phone ownership was identified as a risk factor for healthcare-associated neonatal sepsis in our population. This may be explained if cell phone ownership is used as a corollary for income status, and families who own cell phones may have a higher income/education backgrounds which support advocacy for hospitalized newborns to receive care. However, cell phone ownership could also provide a potential fomite source for infection transmission, especially if parents bring cell phones to all areas of their environment, including bathrooms and cleaning areas. If this mechanism were present in our study, it would be consistent with cell phone contamination and sepsis risk found in other SSA studies.^29^

A recent case control study examined predictors of neonatal sepsis at two referral hospitals in Northwest Ethiopia and showed that poor prenatal care utilization, maternal fever during delivery, prolonged rupture of membranes, and meconium-stained fluid were all associated with neonatal sepsis.^30^ Unlike our investigation, this study did not compare sepsis risk across units or hospital care environments. Other Ethiopian studies corroborate that maternal clinical factors contribute to sepsis risk. A recent cross-sectional study in Amhara identified a strong association between maternal urinary tract infections and neonatal sepsis (adjusted odds ratio (aOR)=6.26).^31^ Other factors such as intrapartum fever (adjusted hazard ratio (AHR) 14.5), maternal chorioamnionitis (AHR 5.7), maternal urinary tract infection (AHR 7.0) and prolonged rupture of membranes (AHR 3.4) predicted neonatal mortality from sepsis.^32^ Of note, all these studies included both home and healthcare facility births, while our study only predicted AMR in healthcare facility births.

Approximately 80% of all healthcare-associated neonatal sepsis cases in the two study hospitals were associated with *Staphylococcus aureus, Klebsiella* spp., *E. coli*, and CoNS. Infants born at the referral hospital were three-fold more likely to develop CoNS infections when compared to the general hospital. Similarly, infants born at the referral hospital were twice as likely to develop infection with *Klebsiella* spp. Systematic reviews have cited *S. aureus, Klebsiella* spp, and *E. coli* as the etiologic agents of 55% of all sepsis cases in LMICs,^11^^,^^17^ and specifically in Ethiopia.^33^, ^34^, ^35^, ^36^ Our findings support the prevalence of these etiologic agents. Coagulase-negative *staphylococci* (CoNS) are part of normal human skin flora and have historically been considered culture contaminants. However, CoNS have emerged as an increasingly frequently isolated pathogen in high-income countries, particularly in the context of healthcare-associated infections, LBW infants, and longer length of stays in the hospital.^37^ While important in high income settings, CoNS less often causes neonatal sepsis in lower income contexts compared to gram negative organisms.^38^ The high prevalence of CoNS in septic infants in this study highlights the increasing concern for CoNS as an emerging pathogen.

AMR patterns in the clinical isolates point to the impact of the environment on the incidence of healthcare-associated neonatal sepsis. Among newborns with culture-confirmed sepsis, there were differences in resistance patterns by facility and birthweight. Overall, 86.4% of all infants had organisms resistant to one or more antibiotics (Table 1). This exceeds previously reported rates of resistant neonatal infections.^8^ In our population, this varied little by birthweight (84.9% for LBW versus 88.9%) and moderately by facility (94.1% at the general hospital versus 80.6%). Because AMR is a multifactorial issue, it is difficult to identify a specific reason for this difference. However, given the significant difference in prevalence between the two sites, this raises questions about the roles of the physical environment and water, sanitation and hygiene (WASH) infrastructure and the infection prevention and control practices (IPC) and human behavior play is newborn sepsis risk.

Despite efforts to identify neonates developing sepsis early in their course and intervening quickly, AMR will continue to thwart these efforts if not mitigated. Determining which neonates are infected with organisms resistant to therapy is vital in rectifying this issue, and this starts with identification of infected neonates. In our study, the empiric clinical diagnosis of sepsis was ultimately determined by the clinician based on the presence of signs and symptoms concerning for sepsis. This may introduce bias in our study based on the clinical suspicion of sepsis. A uniform clinical diagnosis of neonatal sepsis does not currently exist, recalling an important need for early identification tools in LMIC settings where cultures may not be easily accessible. The final diagnosis of sepsis hinges on a positive culture result, which underscores the importance of laboratory capacity at global hospitals—particularly those with a high volume of newborn care. Increasing laboratory capability at healthcare facilities in LMICs will be crucial in aiding the accurate identification of resistant organisms. However, in the absence of powerful, targeted sepsis prevention strategies, organism and resistance identification alone will be a weak intervention. Rather, sepsis prevention—particularly among vulnerable LBW infants is urgently needed. One systematic review proposes a four-pronged approach to address neonatal healthcare-associated infections in LMICs: “promotion of colonization with normal flora; prevention of colonization with pathogens; maintenance of skin integrity; and healthcare-associated infection surveillance, education, and advocacy”.^39^ All of these solutions can be designed or strengthened by interdisciplinary implementation teams and local ministry of health partnerships.

This study provides a practical tool for clinicians who diagnose healthcare-associated neonatal sepsis in LMIC contexts similar to Amhara. The marginal effects analysis highlights the most notable risk factors and illustrates the additive nature of individual risk factors for sepsis. For clinicians, knowledge of the presence or absence of one or more of these risk factors can be useful in risk stratifying patients in real time and tailoring treatment or prevention interventions based on risk. The post-hoc marginal effects analysis using the MLR model identified a more nuanced sepsis-risk prediction with 78% risk variability using four readily identifiable variables at birth.

While neonatal sepsis remains a significant concern in LMIC hospitals, it is imperative to better understand the physical environment and infection prevention and control (IPC) practices in which this risk is occurring. Our findings suggest the healthcare facility itself is an important risk factor for healthcare-associated neonatal sepsis. Further investigation regarding the facility environment, patient density, WASH infrastructure, environmental cleaning, and IPC practices will be important to further elucidate the effect of these factors.

## Conclusion

This study examines the burden of healthcare-associated neonatal sepsis in Amhara, Ethiopia, identifies risk factors for healthcare-associated neonatal sepsis, and emphasizes the high rate of AMR in these infections of the neonatal population. The risk of healthcare-associated neonatal sepsis remains high in LMIC facilities, and the predominance of AMR infections indicates that more emphasis needs to be focused on infection prevention because of the high likelihood of treatment failure and mortality.

The findings of this study could provide evidence to guide advocacy for building hospital laboratory capacity in LMIC healthcare facilities to rapidly identify causative organisms and enable tailored treatment based on AMR profiles, as this may be a key element in decreasing the burden of disease and mortality. Additionally, rigorous examination of empiric therapy regimens for healthcare-associated neonatal sepsis must be done to assess their efficacy. Further investigation of AMR in other areas of sub-Saharan Africa is imperative to further characterize the scope of this threat among neonates.

## CRediT authorship contribution statement

**Nadine Najjar:** Writing – review & editing, Writing – original draft, Formal analysis. **Abebe Gebremariam Gobezayehu:** Writing – review & editing, Supervision, Resources, Project administration, Methodology, Funding acquisition, Formal analysis, Conceptualization. **Joseph Hopkins:** Visualization, Validation, Investigation, Data curation. **Heran Biza:** Validation. **Habib Yakubu:** Project administration, Methodology, Data curation. **Lindsay Denny:** Project administration, Investigation, Data curation. **Mulusew Lijalem:** Project administration, Methodology, Conceptualization. **Lamesgin Alamineh Endalamaw:** Validation, Software, Methodology, Data curation, Conceptualization. **Yakob S. Ahmed:** Writing – review & editing, Data curation. **Taye Zeru Tadege:** Data curation. **Mekuanint Wasihun Endalew:** Data curation. **Yichalal Endayehu Tafere:** Data curation. **Gurmesa Tura Debelew:** Writing – review & editing, Formal analysis. **Erin Stone:** Writing – review & editing. **Christine L. Moe:** Writing – review & editing, Validation, Supervision, Resources, Funding acquisition, Conceptualization. **John N. Cranmer:** Writing – review & editing, Writing – original draft, Visualization, Supervision, Software, Resources, Methodology, Funding acquisition, Formal analysis, Data curation, Conceptualization.

## Declaration of competing interest

The authors declare that they have no known competing financial interests or personal relationships that could have appeared to influence the work reported in this paper.

## Appendix Supplementary materials

Image, application 1

## References

[1] Sharrow D., Hug L., You D., Alkema L., Black R., Cousens S., Croft T., Gaigbe-Togbe V., Gerland P., Guillot M. (2022). Global, regional, and national trends in under-5 mortality between 1990 and 2019 with scenario-based projections until 2030: a systematic analysis by the UN inter-agency group for child mortality estimation. Lancet Glob Health.

[2] Lawn J.E., Blencowe H., Oza S., You D., Lee A.C., Waiswa P., Lalli M., Bhutta Z., Barros A.J., Christian P. (2014). Every newborn: progress, priorities, and potential beyond survival. Lancet.

[3] Wang H., Liddell C.A., Coates M.M., Mooney M.D., Levitz C.E., Schumacher A.E., Apfel H., Iannarone M., Phillips B., Lofgren K.T. (2014). Global, regional, and national levels of neonatal, infant, and under-5 mortality during 1990-2013: a systematic analysis for the global burden of disease study 2013. Lancet.

[4] Fleischmann-Struzek C., Goldfarb D.M., Schlattmann P., Schlapbach L.J., Reinhart K., Kissoon N. (2018). The global burden of paediatric and neonatal sepsis: a systematic review. Lancet Respir Med.

[5] Milton R., Gillespie D., Dyer C., Taiyari K., Carvalho M.J., Thomson K., Sands K., Portal E.A.R., Hood K., Ferreira A. (2022). Neonatal sepsis and mortality in low-income and middle-income countries from a facility-based birth cohort: an international multisite prospective observational study. Lancet Glob Health.

[6] Ethiopian Public Health Institute - EPHI, Federal Ministry of Health - FMoH (2021). Addis Ababa.

[7] Shane A.L., Sanchez P.J., Stoll B.J. (2017). Neonatal sepsis. Lancet.

[8] Zaidi A.K., Huskins W.C., Thaver D., Bhutta Z.A., Abbas Z., Goldmann D.A. (2005). Hospital-acquired neonatal infections in developing countries. Lancet.

[9] Wynn J.L., Wong H.R., Shanley T.P., Bizzarro M.J., Saiman L., Polin R.A. (2014). Time for a neonatal-specific consensus definition for sepsis. Pediatr Crit Care Med.

[10] Williams P.C.M., Isaacs D., Berkley J.A. (2018). Antimicrobial resistance among children in sub-Saharan Africa. Lancet Infect Dis.

[11] Okomo U., Akpalu E.N.K., Le Doare K., Roca A., Cousens S., Jarde A., Sharland M., Kampmann B., Lawn J.E. (2019). Aetiology of invasive bacterial infection and antimicrobial resistance in neonates in sub-Saharan Africa: a systematic review and meta-analysis in line with the STROBE-NI reporting guidelines. Lancet Infect Dis.

[12] (2013). Pocket Book of Hospital Care for Children.

[13] Huynh B.T., Padget M., Garin B., Herindrainy P., Kermorvant-Duchemin E., Watier L., Guillemot D., Delarocque-Astagneau E. (2015). Burden of bacterial resistance among neonatal infections in low income countries: how convincing is the epidemiological evidence?. BMC Infect Dis.

[14] Wen S.C.H., Ezure Y., Rolley L., Spurling G., Lau C.L., Riaz S., Paterson D.L., Irwin A.D. (2021). Gram-negative neonatal sepsis in low- and lower-middle-income countries and WHO empirical antibiotic recommendations: a systematic review and meta-analysis. PLoS Med.

[15] Williams P.C., Qazi S.A., Agarwal R., Velaphi S., Bielicki J.A., Nambiar S., Giaquinto C., Bradley J., Noel G.J., Ellis S. (2022). Antibiotics needed to treat multidrug-resistant infections in neonates. Bull World Health Organ.

[16] (2022). Antimicrobial Resistance C: global burden of bacterial antimicrobial resistance in 2019: a systematic analysis. Lancet.

[17] Downie L., Armiento R., Subhi R., Kelly J., Clifford V., Duke T. (2013). Community-acquired neonatal and infant sepsis in developing countries: efficacy of WHO's currently recommended antibiotics–systematic review and meta-analysis. Arch Dis Child.

[18] Medhanyie A.A., Alemu H., Asefa A., Beyene S.A., Gebregizabher F.A., Aziz K., Bhandari N., Beyene H., Brune T., Chan G. (2019). Kangaroo Mother Care implementation research to develop models for accelerating scale-up in India and Ethiopia: study protocol for an adequacy evaluation. BMJ Open.

[19] Mony P.K., Tadele H., Gobezayehu A.G., Chan G.J., Kumar A., Mazumder S., Beyene S.A., Jayanna K., Kassa D.H., Mohammed H.A. (2021). Scaling up kangaroo mother care in Ethiopia and India: a multi-site implementation research study. BMJ Glob Health.

[20] Gobezayehu A.G., Lijalem M., Endalamaw L.A., Mohammad H., Beyene T., Mekonnen T.B., Abay G.G., Sibley L.M., Cranmer J.N. (2023). Creation of a globally informed and locally relevant KMC implementation model for population-impact in Amhara, Ethiopia. Acta Paediatr.

[21] Harris P.A., Taylor R., Thielke R., Payne J., Gonzalez N., Conde J.G. (2009). Research electronic data capture (REDCap)–a metadata-driven methodology and workflow process for providing translational research informatics support. J Biomed Inform.

[22] Harris P.A., Taylor R., Minor B.L., Elliott V., Fernandez M., O'Neal L., McLeod L., Delacqua G., Delacqua F., Kirby J. (2019). The REDCap consortium: building an international community of software platform partners. J Biomed Inform.

[23] Assemie M.A., Alene M., Yismaw L., Ketema D.B., Lamore Y., Petrucka P., Alemu S. (2020). Prevalence of neonatal sepsis in Ethiopia: a systematic review and meta-analysis. Int J Pediatr.

[24] Belachew A., Tewabe T. (2020). Neonatal sepsis and its association with birth weight and gestational age among admitted neonates in Ethiopia: systematic review and meta-analysis. BMC Pediatr.

[25] Muhe L.M., McClure E.M., Nigussie A.K., Mekasha A., Worku B., Worku A., Demtse A., Eshetu B., Tigabu Z., Gizaw M.A. (2019). Major causes of death in preterm infants in selected hospitals in Ethiopia (SIP): a prospective, cross-sectional, observational study. Lancet Glob Health.

[26] Report on the burden of endemic health care-associated infection worldwide. In.; 2011.

[27] Allegranzi B., Bagheri Nejad S., Combescure C., Graafmans W., Attar H., Donaldson L., Pittet D. (2011). Burden of endemic health-care-associated infection in developing countries: systematic review and meta-analysis. Lancet.

[28] Annual epidemiological report on communicable diseases in Europe 2008. In. Stockholm; 2008.

[29] Aliyo A., Bekele C., Gemechu T., Dedecha W., Getachew M. (2025). Mobile phone bacterial contaminations, associated factors and antimicrobial susceptibility pattern of bacteria isolates from health professionals' working in public health facilities of West Guji zone, Southern Ethiopia. BMJ Open Qual.

[30] Akalu T.Y., Gebremichael B., Desta K.W., Aynalem Y.A., Shiferaw W.S. (2020). Alamneh YM: predictors of neonatal sepsis in public referral hospitals, Northwest Ethiopia: a case control study. PLoS One.

[31] Agnche Z., Yenus Yeshita H., Abdela Gonete K. (2020). Neonatal sepsis and its associated factors among neonates admitted to Neonatal intensive care units in primary hospitals in Central Gondar Zone, Northwest Ethiopia, 2019. Infect Drug Resist.

[32] Dessu S., Habte A., Melis T., Gebremedhin M. (2020). Survival status and predictors of mortality among newborns admitted with neonatal sepsis at public hospitals in Ethiopia. Int J Pediatr.

[33] Shitaye D., Asrat D., Woldeamanuel Y., Worku B. (2010). Risk factors and etiology of neonatal sepsis in Tikur Anbessa University Hospital, Ethiopia. Ethiop Med J.

[34] Worku E., Fenta D.A., Ali M.M. (2022). Bacterial etiology and risk factors among newborns suspected of sepsis at Hawassa, Ethiopia. Sci Rep.

[35] Gashaw M., Ali S., Berhane M., Tesfaw G., Eshetu B., Workneh N., Seeholzer T., Froeschl G., Kroidl A., Wieser A. (2024). Neonatal sepsis due to multidrug-resistant bacteria at a tertiary teaching hospital in Ethiopia. Pediatr Infect Dis J.

[36] Sherif M., Abera D., Desta K. (2023). Prevalence and antibiotic resistance pattern of bacteria from sepsis suspected neonates at St. Paul's Hospital Millennium Medical College, Addis Ababa, Ethiopia. BMC Pediatr.

[37] Dong Y., Speer C.P. (2014). The role of Staphylococcus epidermidis in neonatal sepsis: guarding angel or pathogenic devil?. Int J Med Microbiol.

[38] Russell N., Barday M., Okomo U., Dramowski A., Sharland M., Bekker A. (2024). Early-versus late-onset sepsis in neonates - time to shift the paradigm?. Clin Microbiol Infect.

[39] Dramowski A., Aucamp M., Beales E., Bekker A., Cotton M.F., Fitzgerald F.C., Labi A.K., Russell N., Strysko J., Whitelaw A. (2022). Healthcare-associated infection prevention interventions for neonates in resource-limited settings. Front Pediatr.

