# Supplementary material for: Risk prediction of healthcare-associated neonatal sepsis in Amhara, Ethiopia: A prospective cohort study
Source: Glob Pediatr. 2025 Sep;13:None. doi: 10.1016/j.gpeds.2025.100268 (PMC12396969; doi:10.1016/j.gpeds.2025.100268)
Supplement: Supplementary file 1 [file mmc1.docx]

**Supplement Table 1: Participant Characteristics by Study Hospital**

|  | **Hospital** | | |  |
| --- | --- | --- | --- | --- |
|  | **Total** | **General** | **Referral** | **p-value** |
| **Clinical Factors** |  |  |  |  |
| **Participants**  % (n) | **100.0**  (605)^4^ | **44.1**  (267) | **54.1**  (327) |  |
| **Birth weight** |  |  |  |  |
| Grams (g)  (Median (IQR)) | **2800**  (2300 - 3060) | **2800**  (2400 - 3080) | **2800**  (1980 - 3000) | 0.122^1^ |
| Birthweight  % (n) |  |  |  |  |
| <1500 grams | **4.5**  (26) | **2.3**  (6) | **6.3**  (20) | 0.004^1^ |
| 1500 - <2000 | **15.9**  (92) | **11.5**  (30) | **19.4**  (62) |  |
| 2000 - <2500 | **14.2**  (82) | **15.0**  (39) | **13.5**  (43) |  |
| >2500 | **65.5**  (379) | **71.2**  (185) | **60.8**  (194) |  |
| **Antenatal Care Visits (ANC)** |  |  |  |  |
| Number of ANC visits  (median (IQR)) | **4**  (3-4) | **4**  (3-4) | **4**  (4-5) | 0.001^1^ |
| > ANC (% (n)) | **96.6**  (572) | **94.7**  (252) | **98.2**  (320) | 0.037^3^ |
| ANC - 4 completed visits (% (n)) | **67.0**  (397) | **53.9**  (144) | **77.7**  (254) | 0.000^1^ |
| **Delivery Type** (% (n)) |  |  |  |  |
| Vaginal, normal | **96.5**  (570) | **95.1**  (252) | **97.6**  (318) | 0.207^3^ |
| Assisted vaginal (forceps) | **1.5**  (9) | **1.9**  (5) | **1.2**  (4) |  |
| Caesarean section (c-section) | **2.0**  (12) | **3.0**  (8) | **1.2**  (4) |  |
| Twin birth (% (n)) | **3.4**  (20) | **2.3**  (6) | **4.3**  (14) | 0.252^3^ |
| **Obstetric History** |  |  |  |  |
| Total pregnancies (gravida)  (Median (IQR)) | **2**  (1-3) | **2**  (1-3) | **2**  (1-3) | 0.933^1^ |
| Previous preterm births (% (n))  1 | **16.2** | **10.9** | **20.6** | 0.005^2^ |
| >2 | **4.1** | **3.8** | **4.3** |  |
| Total living children  (Median (IQR)) | **2**  (1-3) | **2**  (1-3) | **2**  (1-3) | 0.358^1^ |
| Total previous births  (Median (IQR)) | **2**  (1-3) | **2**  (1-3) | **2**  (1-3) | 0.337^1^ |
| History of any deceased infant (% (n)) | **4.6** | **4.1** | **4.9** | 0.647^2^ |
| Previous number of LBW births  (% (n))  1 | **18.7** | **11.9** | **24.2** | 0.000^2^ |
| >2 | **3.2** | **2.6** | **3.6** |  |
| **Social Factors** |  |  |  |  |
| Zone of residence^5^ (% (n)) |  |  |  |  |
| South Gondar | **44.7**  (265) | **98.9**  (263) | **0.6**  (2) | 0.000^3^ |
| Bahir Dar City | **36.9**  (219) | **0.0**  (0) | **67.0**  (219) |  |
| West Gojam | **18.4**  (109) | **1.1**  (3) | **32.4**  (106) |  |
| Household size  (Median (IQR)) | **3**  (2-4) | **3**  (2-4) | **3**  (2-4) | 0.079^1^ |
| Own Home (% (n)) | **77.0**  (451) | **59.9**  (160) | **91.2**  (291) | 0.000^2^ |
| Own cell phone (% (n)) | **77.4**  (442) | **53.4**  (135) | **96.5**  (307) | 0.000^2^ |
| Household income  (in 1000 birr)  (Median (IQR)) | **30**  (20-46) | **22**  (18-30) | **40**  (30-54) | 0.000^1^ |
| Income per household member  (Median (IQR)) | **9600**  (6,250-15,666) | **7500**  (5,000-10,000) | **14,000**  (8,500-20,000) | 0.000^1^ |
| Religious affiliation (% (n)) |  |  |  |  |
| Christian orthodox | **96.3**  (570) | **98.9**  (263) | **94.2**  (307) | 0.006^3^ |
| Muslim | **3.0**  (18) | **1.1**  (3) | **4.6**  (15) |  |
| Christian Protestant | **0.7**  (4) | **0.0**  (0) | **1.2**  (4) |  |
| Ethnicity (% (n))  Amhara | **99.7**  (590) | **99.6**  (266) | **99.7**  (324) | 1.000^3^ |
| Other | **0.3**  (2) | **0.4**  (1) | **0.4**  (1) |  |
| **Parent Factors** |  |  |  |  |
|  |  |  |  |  |
| Maternal Ethnicity, Amhara (% (n)) | **99.7**  (590) | **99.7**  (266) | **99.7**  (324) | 0.699^3^ |
| Maternal Age, years (median (IQR)) | **27**  (24-30) | **26**  (23-30) | **27**  (24-30) | 0.229^1^ |
| Maternal Education (% (n)) |  |  |  |  |
| None | **8.1**  (35) | **4.1**  (7) | **10.6**  (28) | 0.000^2^ |
| Primary | **39.6**  (172) | **45.3**  (77) | **36.0**  (95) |  |
| Secondary | **27.7**  (120) | **17.7**  (30) | **34.1**  (90) |  |
| Technical-vocational | **16.1**  (70) | **21.2**  (36) | **12.9**  (34) |  |
| Degree | **8.5**  (37) | **11.8**  (20) | **6.4**  (17) |  |
| Maternal Literacy (% (n)) | **73.9**  (438) | **64.7**  (170) | **82.2**  (268) | 0.000^2^ |
| Maternal Occupation (% (n)) |  |  |  |  |
| Homemaker | **52.2**  (309) | **40.6**  (108) | **61.6**  (201) | 0.000^3^ |
| Farmer | **19.1**  (113) | **24.8**  (66) | **14.4**  (47) |  |
| Professional | **9.1**  (54) | **9.0**  (24) | **9.2**  (30) |  |
| Small Trade | **6.9**  (41) | **11.3**  (30) | **3.4**  (11) |  |
| Merchant | **2.0**  (12) | **2.6**  (7) | **1.5**  (5) |  |
| Skilled labor | **6.9**  (41) | **6.4**  (17) | **7.4**  (24) |  |
| Unskilled labor | **3.2**  (19) | **4.1**  (11) | **2.5**  (8) |  |
| Religious leader | **0.2**  (1) | **0.4**  (1) | **0.0**  (0) |  |
| Student | **0.3**  (2) | **0.8**  (2) | **0.0**  (0) |  |
|  |  |  |  |  |
| Paternal Age, years (Median (IQR)) | **30**  (28-35) | **31**  (22-36) | **30**  (28-35) | 0.076^1^ |
| Paternal Education (% (n)) |  |  |  |  |
| None | **7.5**  (35) | **6.3**  (13) | **8.4**  (22) | 0.000^2^ |
| Primary | **27.6**  (129) | **31.1**  (64) | **24.8**  (65) |  |
| Secondary | **32.3**  (151) | **21.8**  (45) | **40.5**  (106) |  |
| Technical-vocational | **13.0**  (61) | **19.9**  (41) | **7.6**  (20) |  |
| Degree | **19.7**  (92) | **20.9**  (43) | **18.7**  (49) |  |
| Paternal Literacy (% (n)) | **70.1**  (469) | **80.7**  (263) | **77.2**  (206) | 0.294^2^ |
| Paternal Occupation (% (n)) |  |  |  |  |
| Farmer | **29.3**  (173) | **33.3**  (86) | **26.8**  (87) | 0.000^1^ |
| Skilled | **25.9**  (153) | **18.1**  (48) | **32.3**  (105) |  |
| Professional | **16.2**  (96) | **18.8**  (50) | **15.2**  (46) |  |
| Unskilled | **11.8**  (70) | **15.7**  (39) | **9.5**  (31) |  |
| Merchant | **11.2**  (66) | **7.9**  (21) | **13.9**  (45) |  |
| Small Trade | **3.1**  (18) | **4.9**  (13) | **1.5**  (5) |  |
| Religious leader | **1.7**  (10) | **2.3**  (6) | **1.2**  (4) |  |
| Student | **0.3**  (2) | **0.4**  (1) | **0.3**  (1) |  |
| Unemployed | **0.3**  (2) | **0.8**  (2) | **0.0**  (0) |  |
| Police | **0.2**  (1) | **0.0**  (0) | **0.3**  (1) |  |
|  |  |  |  |  |
|  |  |  |  |  |
| Parental age gap, years (Dad-Mom)  (Median (IQR)) | **5**  (3-7) | **5**  (3-6) | **5**  (3-7) | 0.071^1^ |
| Number of parents literate (% (n)) |  |  |  |  |
| 0 literate | **15.7**  (93) | **20.2**  (54) | **12.0**  (39) | 0.001^3^ |
| 1 literate | **15.7**  (93) | **18.7**  (50) | **13.2**  (43) |  |
| 2 literate | **68.6**  (407) | **61.1**  (163) | **74.6**  (244) |  |

^1^ Kruskal Wallis H-test

^2^ Pearson’s Chi-squared test of independence

^3^ Fisher’s exact test

^4^ 605 infants were recruited at baseline; 1.8% did not have the recruiting facility reported (n=11).

^5^ A zone is the second highest level of administrative structure after a region. The average population of a zone in Amhara region is about 1.9 million. Each administrative zone on average has about 12 districts/woredas under it. Each woreda/district has an estimated population of 160,000.
